# Comprehensive analysis of metabolomics on flesh quality of yellow catfish (*Pelteobagrus fulvidraco*) fed plant-based protein diet

**DOI:** 10.3389/fnut.2023.1166393

**Published:** 2023-04-14

**Authors:** Xue Li, Shidong Wang, Muzi Zhang, Haibo Jiang, Yunxia Qian, Rixin Wang, Ming Li

**Affiliations:** ^1^School of Marine Sciences, Ningbo University, Ningbo, China; ^2^College of Animal Science, Guizhou University, Guiyang, China

**Keywords:** flesh quality, myofiber development, metabolomics, flavor amino acids, *Pelteobagrus fulvidraco*

## Abstract

**Background:**

To investigate the mechanism of plant protein components on nutritional value, growth performance, flesh quality, flavor, and proliferation of myocytes of yellow catfish (*Pelteobagrus fulvidraco*).

**Methods:**

A total of 540 yellow catfish were randomly allotted into six experimental groups with three replicates and fed six different diets for 8 weeks.

**Results and Conclusions:**

The replacement of fish meal with cottonseed meal (CM), sesame meal (SEM), and corn gluten meal (CGM) in the diet significantly reduced growth performance, crude protein, and crude lipid, but the flesh texture (hardness and chewiness) was observably increased. Moreover, the flavor-related amino acid (glutamic acid, glycine, and proline) contents in the CM, SEM, and CGM groups of yellow catfish muscle were significantly increased compared with the fish meal group. The results of metabolomics showed that soybean meal (SBM), peanut meal (PM), CM, SEM, and CGM mainly regulated muscle protein biosynthesis by the variations in the content of vitamin B6, proline, glutamic acid, phenylalanine, and tyrosine in muscle, respectively. In addition, Pearson correlation analysis suggested that the increased glutamic acid content and the decreased tyrosine content were significantly correlated with the inhibition of myocyte proliferation genes. This study provides necessary insights into the mechanism of plant proteins on the dynamic changes of muscle protein, flesh quality, and myocyte proliferation in yellow catfish.

## 1. Introduction

To achieve the cost reduction of aquaculture feed, it is necessary to use cheaper plant protein sources to replace the expensive fish meal partially or completely. Meanwhile, dietary plant protein sources are considered to be the primary factors affecting the flesh quality of fish (1, 2). Among various plant protein sources, researchers have attracted great attention from soybean meal because of its stable nutrient composition, relatively balanced amino acid composition, and reasonable price (3, 4). A previous study showed that adding soybean protein to diets can significantly improve the hardness and chewiness of flesh in European bass (*Dicentrarchus labrax*) (5). The peanut meal is abundant in amino acids, but the amino acid composition is unbalanced, and it shows that the content of methionine, lysine, and tryptophan is relatively scarce. A previous study has shown that the use of up to 25% peanut meal has no adverse effects on the growth, feed efficiency, and body composition of channel catfish (6). However, the addition of a high level of peanut meal in the diet will not only delay the growth performance and development of crucian carp but also induce hazards to health and flesh quality (7). The cottonseed meal has higher protein content and better flavor and was considered to be a better protein source for fish; however, it contained gossypol which was an anti-nutritional factor (8). A study has shown that high fish meal replacement by cottonseed protein concentrate negatively affects growth and flesh quality in largemouth bass (*Micropterus salmoides*) (9). The sesame meal was rich in methionine and tryptophan, but the contents of other amino acids are far lower than the fish meal or soybean meal. Excessive use of sesame meal in the diet can reduce the crude protein content and change the muscle amino acid composition of muscle in European sea bass (*Dicentrarchus labrax*), thereby affecting the flavor and taste of the flesh (10). The corn gluten meal had a higher protein, vitamin B, and vitamin E content, lower fiber content, and anti-nutritional factors (11). However, the composition of amino acids in corn gluten meal was unbalanced, and lysine and arginine were deficient. A previous study has shown that the amount of muscle umami and sweet amino acids in turbot (*Psetta maxima*) were decreased by corn gluten meal (12). To sum up, a large number of studies had shown that plant protein components can affect the flesh quality of cultured fish, but the physiological mechanism was ambiguous.

Hygienic quality, nutritional quality, sensory quality, and suitability of meat are defined as four terminologies to describe the quality of meat (13). The nutritional value of fish mainly includes the contents of protein (essential amino acids), lipids (essential fatty acids), and carbohydrates, while the flavor of flesh depended on some flavor substances, such as umami amino acids and fatty acids (13). Glutamic acid and aspartic acid can directly increase the umami taste of flesh, and proline, threonine, serine, glycine, and alanine can directly increase the sweetness of flesh in fish (14). In addition, the texture of fish is directly related to the physiological state of myocytes, smaller muscle fiber diameter combined with higher collagen content represents higher hardness and better taste (15). The differentiation and growth of muscle fiber cells, as well as the hyperplasia and hypertrophy of muscle, are controlled by a series of myogenic growth transcription factors and signaling pathways, including myogenic regulatory factors (*MRFs*) and myostatin (*MSTN*) in fish muscle (16). Myogenic differentiation (*MyoD*) and myogenin (*MyoG*) are involved in the regulation of muscle formation and differentiation. Determination of the gene expression levels of *MyoD* and *MyoG* can be used to initially evaluate the developmental state of muscle (5, 17). *MRFs* and *MSTN* regulated the proliferation and differentiation of myogenic progenitor cells (MPCs) and enhance muscle growth by activating myogenic-related signaling pathways have been well documented in fish (18). A previous study showed that amino acid imbalance plant protein enhanced the morphology and development of myocytes and improved the taste and flesh quality of yellow river carp (*Cyprinus carpio*), which is mainly due to smaller muscle fiber diameter and the interspace between the myofilaments were filled with more intramuscular collagen (19). Metabolites in muscle play a crucial role in flavor and aroma characteristics, nutritional value, degradation, and biosynthesis of protein in fish. Metabolomics can detect variations in metabolic profiles of various low molecular weight metabolites, such as amino acids, lipids, and sugars (20, 21). Gene expression can provide information on the expression activity of enzymes and functional proteins related to the proliferation and formation of myocytes (22).

Yellow catfish (*Pelteobagrus fulvidraco*) is one of the most important aquaculture species in major freshwater river basins of China (23). Yellow catfish has the characteristics of delicious and tender meat, high protein, and less intermuscular bone, which is especially suitable for young children’s consumption. However, due to the limitation of aquaculture conditions under the pond culture mode, the flesh quality of yellow catfish is often softer and worse than that of wild yellow catfish. The market price and consumer acceptance of yellow catfish have also decreased. In recent years, with the gradual increase of requirements of consumers for the quality of aquatic products, effectively improving the flesh quality and flavor of cultured yellow catfish has become a serious issue to be solved urgently. It is an efficient and eco-friendly approach to improve the flesh quality and flavor of yellow catfish by utilizing a variety of inexpensive plant protein sources. In this study, five common plant protein sources (soybean meal, SBM; peanut meal, PM; cottonseed meal, CM; sesame meal, SEM; corn gluten meal, CGM) were used to replace 50% fish meal (FM), respectively. Then, the growth performance, body composition, texture parameters, and muscle amino acid composition of yellow catfish were evaluated through an 8-week growth test, in order to evaluate its potential application value in improving flesh quality. However, no studies have been conducted to investigate the mechanism of plant protein sources on the biosynthesis of muscle protein and proliferation of myocytes in yellow catfish while monitoring growth performance and flesh quality. Therefore, the present study utilized a combination of molecular biology and metabolomics techniques to adequately explore the molecular mechanisms by which different plant proteins affect the proliferation and degradation of muscle proteins. It provides a theoretical basis for further understanding the mechanism of plant proteins regulating the flesh quality of yellow catfish.

## 2. Materials and methods

### 2.1. Experimental diets and composition

Fish meal (420 g/kg) as a protein source was used as a control. Five experimental diets were formulated by substituting 50% of fish meal with SBM, PM, CM, SEM, and CGM (24). The feed was made according to the previous steps of our laboratory (25). The brief operation steps are as follows: Grind all ingredients adequately through a 60 μm mesh sieve, mix all ingredients thoroughly, and add fish oil to the dry mixture. Add enough deionized water to the paste to get the appropriate granulation consistency. The mixture is pressurized by the counter-rotation twin-screw extruder and made into long strips by the 2.5 mm diameter porous mold, and then fractured into granular feed after high-speed rotation. The granular feed was dried at room temperature until the water content was <100 g/kg. Store the dried feed in a zip-lock bag tightly sealed and stored in a refrigerator at a temperature of −20°C (25). The rough material and crude composition of the diets in the experiment were shown in Table 1.

**Table 1:** Formulation of the experimental diets (g/kg dry matter basis).

	FM	SBM	PM	CM	SEM	CGM
Ingredient						
Fish meal	560.00	280.00	280.00	280.00	280.00	280.00
Wheat meal	180.00	180.00	180.00	180.00	180.00	180.00
Soybean meal	0.00	350.00	0.00	0.00	0.00	0.00
Peanut meal	0.00	0.00	360.00	0.00	0.00	0.00
Cottonseed meal	0.00	0.00	0.00	300.00	0.00	0.00
Sesame meal	0.00	0.00	0.00	0.00	320.00	0.00
Corn gluten meal	0.00	0.00	0.00	0.00	0.00	300.00
Casein	10.00	50.00	10.00	10.00	50.00	10.00
Fish oil	10.00	28.00	28.00	28.00	28.00	28.00
Soybean lecithin	10.00	10.00	10.00	10.00	10.00	10.00
Vitamin mix^a^	10.00	10.00	10.00	10.00	10.00	10.00
Mineral mix^b^	5.00	5.00	5.00	5.00	5.00	5.00
Monocalcium phosphate	20.00	20.00	20.00	20.00	20.00	20.00
Choline chloride	5.00	5.00	5.00	5.00	5.00	5.00
Cellulose	169.50	41.50	71.5	131.5	71.5	131.5
Sodium carboxymethylcellulose	20.00	20.00	20.00	20.00	20.00	20.00
Ethoxyquin	0.50	0.50	0.50	0.50	0.50	0.50
Proximate composition						
Crude protein	420.46	422.35	421.76	423.63	424.61	427.66
Crude lipid	119.01	115.72	120.49	115.02	121.89	116.19
Ash	73.61	73.53	76.84	73.93	74.60	74.09
Moisture	41.69	41.81	42.10	40.62	41.95	40.10

a Vitamin premix (g/kg diet): Vitamin A, 0.032; Vitamin D, 0.005; Vitamin E, 0.24; Vitamin K, 0.01; Vitamin B_1_, 0.025; Vitamin B_2_, 0.045; Nicotinic acid, 0.2; Vitamin B_6_, 0.02; Biotin, 0.06; Inosito, 1.8; Calcium pantothenate, 0.06; Folic acid, 0.02; Vitamin B_12_, 0.01; Vitamin C, 2; Microcrystalline cellulose, 6.29.

b Mineral mix (g/kg premix): CuSO_4_ • 5H_2_O, 0.01; Na_2_SeO_3_, 0.02; MnSO_4_ • H_2_O, 0.045; CoCl_2_ • 6H_2_O, 0.05; ZnSO_4_ • H_2_O, 0.5; Ca (IO_3_)_2_, 0.06; FeSO_4_ • H_2_O, 0.08; MgSO_4_ • 7H_2_O, 1.2; Zeolite powder, 3.485.

### 2.2. Experimental animals

This study was conducted in eighteen cylindrical plastic barrels (diameter of about 0.5 m, height of about 0.9 m), and juvenile yellow catfish (1.67 ± 0.27 g) were obtained from a commercial aquaculture farm (Huzhou, China). Thirty fish were raised in each plastic bucket. Each treatment group was equipped with 3 repeating culture barrels. A PVC pipe with a diameter of 15 cm is installed in each bucket to serve as a shelter, and sufficient aeration is carried out with an air stone. 70% of the total water volume is changed once a week. The water temperature was 28 ± 2°C. The pH was between 7.6 and 8.0, and the dissolved oxygen content was above 4 mg/L. Fish are fed twice a day at 7 a.m. and 7 p.m., respectively. The feeding experiment lasted for 8 weeks.

### 2.3. Sampling

At the end of the feeding experiment, anesthesia was administered with 50 mg/L tricaine methanesulfonate (MS222; Sigma, St Louis, MO, United States) for weighing and counting. Five fish were randomly selected from each barrel and stored at −20°C for crude composition analysis of whole-body proximate composition analysis. Blood samples (three fish in each barrel) were collected and centrifuged for the analysis of serum biochemical indices, and the muscles on both sides, liver and visceral mass were stripped. A portion of muscle samples was isolated from *Vivo* and quickly frozen in liquid nitrogen and stored at −80°C for subsequent q-PCR, and metabolomics analysis. The other muscle samples were fixed with paraformaldehyde and then prepared by hematoxylin and eosin staining methods (18). The muscles of another 5 fresh fish were dissected and the texture analysis was performed in the base immediately.

### 2.4. Analysis of whole-body composition

All experimental diets and fish samples were analyzed for the approximate composition according to standard methods in AOAC (26). The crude protein content was determined by the combustion method using an FP-528 nitrogen analyzer (Leco Corporation, St. Joseph, MI, United States). The crude lipid content was determined by the soxhlet extraction system (2055 Soxtec Avanti; Foss Tecator, Hoganas, Sweden), and ash content was determined by muffle furnace (SX2-4-10N, Yiheng) heating at 550°C for 6 h.

### 2.5. Biochemical analysis

Serum was separated after centrifuging (3,000 r/min, 4°C, 15 min). Strictly followed the operation instructions of commercial enzyme kits (Nanjing Jiancheng Institute of Bioengineering, Nanjing, China) to conduct the mensuration of the contents of total triglyceride (TG), total cholesterol (TC), high-density lipoprotein cholesterol (HDL), and low-density lipoprotein cholesterol (LDL), and the absorbance was determined by the PT-3502C enzyme marker of Putian Xinqiao Technology Co., LTD. (Beijing, China). Finally, the activity and content of each enzyme were calculated according to the formula provided in the instructions (25).

### 2.6. Muscle texture analysis

Muscle masses (20 mm × 10 mm × 10 mm) were dissected from the fish above the lateral line, and texture and shear force tests were performed on the muscle samples using a flat-bottomed cylindrical probe (TA. new plus Texture Analyzer, ISENSO, United States). The probe was loaded with Auto-5 g, and the sample was compressed to 1/2 of the original thickness by two consecutive cycles at a constant speed of 1 mm/s and an interval of 5 s.

### 2.7. Muscle amino acid composition analysis

The determination of muscle amino acid composition was based on the previously established method with some modifications. In short, amino acid composition in fish muscle was determined by an automatic amino acid analyzer (HITACHI, LA8080, Japan). The freeze-dried muscle sample was weighed 40–50 mg (accurate to 0.1 mg), placed in a 50 mL ampoule bottle, and marked. Adding 6 mol/L hydrochloric acid 10 mL quickly sealed the tube and placed the tube into the constant temperature drying oven (110 ± 1) °C for 24 h. Opened the ampoule bottle after cooling, filled miscible liquids into a 150 mL rotary evaporation bottle with ultra-clean water, and vacuum it in the rotary evaporator (60°C) to evaporate until it dry. Rinsed the evaporation bottle with 0.02 mol/L hydrochloric acid for several times and transferred the washing liquid to a 10 mL volumetric bottle. Finally, the sample hydrolysate was fully mixed with 0.02 mol/L hydrochloric acid. Absorbed 2–3 mL hydrolysate and mixed amino acid standard solution of the sample, filtered into an automatic sampling bottle, and amino acid content was detected by an automatic amino acid analyzer (27).

### 2.8. q-PCR analysis

Total RNA was extracted from the muscle tissue of yellow catfish using an RNAiso reagent kit (Takara Bio. Inc., China). Roche LightCycler^®^480 II real-time PCR instrument (Roche Ltd., Switzerland) and SYBR Premix Ex Taq (Takara Bio. Inc., China) were used for qPCR detection. Primer 6 was used to design specific primers for q-PCR (Table 2). The PCR temperature conditions were 95°C for 30 s followed by 40 cycles of 95°C for 20 s, 57°C for 25 s, and 72°C for 25 s. The expression levels were calculated using the 2^−ΔΔCT^ method (28).

**Table 2:** Primers used in this study.

Primer	Primer sequence (5′–3′)	Product length	Login number
*Myod*	F: TATTCCGTTCCCCATCCCCT	208	XM_027173047.2
	R: TTTACACGCCCACAGGAGAC		
*Myf5*	F: GGCTAGAGAAGGTGAACCAC	290	XM_027155249.2
	R: CGCACTCTGACCTTCGTAAC		
*Myog*	F: ACCCGTACTTTTTCCCCGAA	129	XM_027144245.2
	R: CATCCCCACATAGCCCTACC		
*IGF*	F: GCACAACCGTGGCATTGTAG	135	XM_027160377.2
	R: GACGTGTCTGTGTGCCGTT		
*MSTN*	F: GCGCACCAAGAGAGAATCAG	125	XM_027171347.2
	R: AGCGTTTCGGGGCAATAATC		
*mTOR*	F: GCAAACTGCTGGTTGTAGGC	261	XM_027166728.2
	R: TGTGCTCCAGCTCAGTCAAG		
*Rptor*	F: GACATCCACCCAAAAGCCAA	288	XM_047813510.1
	R: TCATTTACATCCTGCCCACG		
*mLST8*	F: CTCCACATTGCTGGCTACCT	252	XM_027137533.2
	R: ACAGCCTTTTGATGCCCACT		
*RPL13*	F: GAGAGTGGCTCGTACCATCG	282	XM_027136566.2
	R: AGACTGGCGAAAGCCTTGAA		
*β-actin*	F: TTCGCTGGAGATGATGCT	136	XM_027148463.2
	R: CGTGCTCAATGGGGTACT		
*GAPDH*	F: TCTGGGGTACACAGAACACC	165	XM_027149217.1
	R: ACTAGGTCACAGACACGGTT		

Reference genes were *β-actin* and *GAPDH*, respectively.

### 2.9. Metabonomic analysis

Homogenate was extracted with tissue extract (75% 9:1 methanol: chloroform, 25% H_2_O) and injected into liquid chromatography-mass spectrometry (LC–MS) system. The LC analysis was performed on a Vanquish UHPLC System (Thermo Fisher Scientific, United States). Chromatography was carried out with an ACQUITY UPLC^®^ HSS T3 (150 × 2.1 mm, 1.8 μm) (Waters, Milford, MA, United States). The column was maintained at 40°C. The flow rate and injection volume were set at 0.25 mL/min and 2 μL, respectively. For LC-ESI (+)-MS analysis, the mobile phases consisted of (C) 0.1% formic acid in acetonitrile (v/v) and (D) 0.1% formic acid in water (v/v). Separation was conducted under the following gradient: 0–1 min, 2% C; 1–9 min, 2%–50% C; 9–12 min, 50%–98% C; 12–13.5 min, 98% C; 13.5–14 min, 98%–2% C; 14–20 min, 2% C. For LC-ESI (−)-MS analysis, the analytes were carried out with (A) acetonitrile and (B) ammonium formate (5 mM). Separation was conducted under the following gradient: 0–1 min, 2%A; 1–9 min, 2%–50%A; 9–12 min, 50%–98%A; 12–13.5 min, 98%A; 13.5–14 min, 98%–2%A; 14–17 min, 2%A (29). Mass spectrometric detection of metabolites was performed on Orbitrap Exploris 120 (Thermo Fisher Scientific, United States) with an ESI ion source. The above experiments are completed in BioNovoGene (Suzhou, China). The MSConvert tool in the Proteowizard software package (V3.0.8789) was used to convert the original mass spectrometry file into mzXML file format. The R XCMS software package was used for peak detection, peak filtering, and peak alignment, and the quantitative list of substances was obtained. Public databases HMDB, Massbank, LipidMaps, MZClound, and KEGG (Kyoto Encyclopedia of Genes and Genomes) were used to identify the substances.

### 2.10. Statistical analysis

All data are expressed as mean ± standard error (SEM). One-way ANOVA was used to evaluate the significant difference between the FM group and SBM, PM, CM, SEM, and CGM groups. All analyses were performed using SPSS 18.0.0 software. There was a significant difference in the probability level determination of *p* < 0.05 across all analyses. Online software MetaboAnalyst^1^ was used for principal component analysis (PCA), differential metabolite analysis, and functional pathway enrichment analysis. The Pearson correlation analysis between metabolites and physiological indexes (crude protein, crude lipid, and expression of genes related to muscle proliferation) was conducted by R package. Pearson-R > 0.8 or Pearson-R < −0.8 and *p* < 0.05 were considered to indicate a significant correlation (30). GraphPad Prism 8.0.2 (San Diego, United States) software was used to complete the presentation of the volcano map of differential metabolite and the bubble maps of the KEGG signal pathway enriched.

## 3. Results

### 3.1. Growth performance and serum biochemical parameter

After 8 weeks of feeding, the body length (BL) in CM and CGM groups was significantly lower than that in the FM group (*p* < 0.05) (Table 3); the body height (BH), weight gain rate (WG), and specific growth rate (SGR) in SBM, PM, CM, SEM, and CGM groups were also significantly lower than those in the FM group (*p* < 0.05). However, the hepatosomatic index (HSI) and feed conversion ratio (FCR) in SBM, PM, CM, SEM, and CGM groups were significantly higher than that in the FM group (*p* < 0.05). Feed consumption (FC) in CM and CGM groups was significantly lower than in the FM group (*p* < 0.05). The viscerasomatic index (VSI) in the SBM group was lower the than FM group (*p* < 0.05). There was no significant difference in condition factor (*CF*) and survival rate (SUR) among the six experimental groups (*p* > 0.05). The serum total cholesterol (TC) content in the PM, CM, SEM, and CGM groups was higher than that in the FM group (*p* < 0.05). The low-density lipoprotein (LDL) content in SEM and CGM groups was significantly lower than that in the FM group, while the high-density lipoprotein (HDL) the content in PM, CM, and SEM groups was significantly higher than FM group (*p* < 0.05).

**Table 3:** The effect of five typical plant proteins partially replacing fish meal on the growth performance and serum biochemical parameters of yellow catfish.

	FM	SBM	PM	CM	SEM	CGM
Growth performance						
BL, cm	9.90 ± 0.15^b^	9.53 ± 0.03^ab^	9.30 ± 0.15^ab^	9.07 ± 0.07^a^	9.20 ± 0.45^ab^	8.90 ± 0.15^a^
BH, cm	2.13 ± 0.03b	1.73 ± 0.03^a^	1.77 ± 0.03^a^	1.67 ± 0.03^a^	1.67 ± 0.03^a^	1.50 ± 0.06^a^
IW, g	49.75 ± 0.28	52.23 ± 0.64	51.73 ± 0.32	53.15 ± 0.44	54.13 ± 0.70	54.47 ± 0.46
FW, g	305.30 ± 13.93^b^	261.10 ± 10.72^ab^	249.30 ± 22.07^a^	220.87 ± 7.32^a^	251.53 ± 21.65^a^	201.93 ± 9.76^a^
WG, %	535.10 ± 21.89^c^	430.20 ± 19.92^b^	402.83 ± 32.83^b^	398.51 ± 7.30^b^	402.79 ± 15.07^b^	307.49 ± 12.60^a^
SGR, %	3.30 ± 0.06^b^	2.98 ± 0.07^a^	2.88 ± 0.12^a^	2.87 ± 0.03^a^	2.88 ± 0.05^a^	2.51 ± 0.05^a^
HSI, %	1.01 ± 0.05^a^	1.77 ± 0.06^b^	1.76 ± 0.10^b^	1.65 ± 0.07^b^	1.78 ± 0.07^b^	2.47 ± 0.11^c^
VSI, %	10.31 ± 0.72^b^	8.14 ± 0.46^a^	8.45 ± 1.23^ab^	8.70 ± 0.20^ab^	8.56 ± 0.10^ab^	8.37 ± 0.06^ab^
*CF*, %	1.08 ± 0.04	1.07 ± 0.04	1.07 ± 0.02	1.19 ± 0.04	1.19 ± 0.16	1.05 ± 0.03
FCR	1.93 ± 0.07^a^	2.31 ± 0.11^b^	2.38 ± 0.16^b^	2.44 ± 0.06^b^	2.33 ± 0.15^b^	2.81 ± 0.14^b^
SUR, %	96.67 ± 3.33	94.45 ± 4.01	95.56 ± 2.94	83.33 ± 1.93	92.22 ± 6.19	91.11 ± 4.84
Serum biochemical parameters						
TG, mmol/L	2.70 ± 0.31	3.09 ± 0.24	2.86 ± 0.38	2.49 ± 0.23	2.45 ± 0.12	2.99 ± 0.50
TC, mmol/L	3.93 ± 0.23^a^	3.85 ± 0.24^a^	4.91 ± 0.12^b^	4.74 ± 0.17^b^	5.26 ± 0.12^b^	5.12 ± 0.21^b^
LDL, mmol/L	5.98 ± 0.36^b^	4.76 ± 0.58^ab^	6.26 ± 0.56^b^	5.04 ± 0.30^ab^	3.59 ± 0.45^a^	3.37 ± 0.27^a^
HDL, mmol/L	2.59 ± 0.25^a^	2.63 ± 0.70^a^	3.39 ± 0.38^b^	3.57 ± 0.39^b^	3.25 ± 0.55^b^	3.05 ± 0.64^ab^

Values are means ± SEM of 3 replications. Values within a row without a common superscript letter (a, b) are different (c indicates a higher value). WG % = Weight gain = 100 × (final weight-initial weight)/initial weight; SGR%·day^−1^ = [In (final weight) − In (initial weight)] × 100∕days, HSI % = Hepatosomatic index = 100 × liver weight, g/body weight, g; VSI% = Viscerasomatic index = 100 × viscerasomatic weight, g/body weight, g; *CF* % = Condition factor = 100 × body weight, g/body length, cm^3^; FCR = feed conversion ratio = feed consumption, g/(final weight-initial weight); SUR % = 100 × number of surviving fish/numbers of dead fish.

### 3.2. Whole-body composition and muscle texture parameter

The crude protein content in PM, CM, SEM, and CGM groups was lower than that in the FM group (*p* < 0.05) (Table 4). The crude lipid content in SBM, CM, SEM, and CGM was also lower than that in the FM group (*p* < 0.05). There was no significant difference in moisture and ash contents (*p* < 0.05). The hardness and chewiness in SBM, CM, SEM, and CGM groups were significantly higher than those in the FM group (*p* < 0.05). The springiness and gumminess in SEM and CGM groups were significantly higher than those in the FM group (*p* < 0.05). The resilience in the SEM group was higher than that in the FM group (*p* < 0.05).

**Table 4:** Effect of five typical plant proteins partially replaces fish meal on the whole-body proximate composition and muscular textural properties in yellow catfish.

Experimental group	FM	SBM	PM	CM	SEM	CGM
Whole-body composition						
Moisture, %, WW	76.60 ± 0.34	76.37 ± 1.13	76.07 ± 1.69	73.95 ± 1.36	75.85 ± 0.42	76.72 ± 0.70
Crude protein, %, DW	56.97 ± 0.24^b^	56.71 ± 0.04^b^	52.75 ± 0.15^a^	53.03 ± 0.38^a^	53.44 ± 0.07^a^	53.99 + 0.24^a^
Crude lipid, %, DW	25.27 ± 0.11^b^	19.54 ± 0.04^a^	25.38 ± 0.37^b^	21.61 ± 0.21^a^	20.85 ± 0.43^a^	18.46 ± 0.78^a^
Ash, %, WW	12.57 ± 0.31	12.50 ± 0.14	12.46 ± 0.12	12.04 ± 0.16	12.54 ± 0.14	12.65 ± 0.61
Texture parameters						
Hardness	17.27 ± 2.24^a^	25.36 ± 4.08^b^	19.47 ± 2.09^a^	27.76 ± 3.15^b^	31.56 ± 3.32^bc^	36.00 ± 1.16^c^
Chewiness	7.68 ± 0.94^a^	12.86 ± 2.11^b^	8.23 ± 0.88^a^	10.94 ± 0.15^b^	18.33 ± 2.37^c^	17.96 ± 0.98^c^
Gumminess	13.01 ± 1.16^a^	18.52 ± 1.76^a^	21.82 ± 3.41^a^	16.35 ± 0.52^a^	24.22 ± 2.62^b^	25.85 ± 1.22^b^
Cohesiveness	0.75 ± 0.02	0.73 ± 0.01	0.72 ± 0.01	0.69 ± 0.01	0.77 ± 0.01	0.72 ± 0.02
Resilience	0.30 ± 0.02^a^	0.33 ± 0.01^a^	0.31 ± 0.02^a^	0.31 ± 0.02^a^	0.42 ± 0.03^b^	0.33 ± 0.02^a^

Values are means ± SEM of 3 replications; Values within a row without a common superscript letter (a, b) are different (c indicates a higher value). WW and DW are the abbreviation of wet weight basis and dry weight basis.

### 3.3. Muscle amino acid content and fatty acid composition

Compared with the FM group, the contents of 16 amino acids detected in the SBM group had no obvious variations (*p* > 0.05) (Table 5). The content of glycine in the PM group was significantly higher than in the FM group, and other amino acids were significantly lower than in the FM group (*p* < 0.05). The contents of glutamic acid, glycine, and proline which belong to flavor amino acids, were significantly increased in the CM group compared with the FM group (*p* < 0.05), and the content of phenylalanine in the CM group was also higher than that in FM group (*p* < 0.05). The contents of phenylalanine, arginine, glutamic acid, glycine, and proline in the SEM group were significantly higher than that in the FM group, while the content of tyrosine was significantly decreased (*p* < 0.05). The contents of methionine, phenylalanine, arginine, glutamic acid, glycine, and proline in the CGM group were significantly higher than those in the FM group, while the content of tyrosine was also significantly decreased compared with the FM group (*p* < 0.05).

**Table 5:** Effect of five typical plant proteins partially replacing fish meal on the amino acid content and fatty acid composition of muscle in yellow catfish.

	FM	SBM	PM	CM	SEM	CGM
Essential amino acids (g/kg dry matter)						
Threonine	37.96 ± 0.41^b^	36.70 ± 0.72^b^	33.45 ± 0.30^a^	39.17 ± 0.68^b^	40.12 ± 1.07^b^	39.65 ± 0.80^b^
Valine	38.81 ± 0.48^b^	37.77 ± 0.74^b^	33.68 ± 0.23^a^	39.88 ± 0.75^b^	40.70 ± 1.26^b^	40.57 ± 0.98^b^
Methionine	22.47 ± 0.71^b^	21.87 ± 0.14^b^	17.93 ± 0.39^a^	24.20 ± 0.74^b^	23.96 ± 0.57^b^	24.39 ± 0.28^c^
Isoleucine	35.75 ± 0.37^b^	34.70 ± 0.55^b^	30.62 ± 0.02^a^	36.71 ± 0.74^b^	37.48 ± 1.27^b^	37.45 ± 0.94^b^
Phenylalanine	35.75 ± 0.67^b^	34.99 ± 0.52^b^	31.15 ± 0.06^a^	37.61 ± 0.66^c^	37.66 ± 1.34^c^	37.24 ± 1.07^c^
Leucine	65.70 ± 0.94^b^	62.86 ± 1.21^b^	55.35 ± 0.12^a^	67.71 ± 1.40^b^	68.16 ± 2.75^b^	67.75 ± 1.58^b^
Histidine	21.68 ± 0.50^b^	20.34 ± 0.34^b^	18.92 ± 0.05^a^	22.24 ± 0.12^b^	21.89 ± 0.82^b^	21.92 ± 0.35^b^
Lysine	78.01 ± 0.82^b^	74.97 ± 1.41^b^	65.48 ± 0.06^a^	79.25 ± 1.75^b^	80.22 ± 3.57^b^	79.78 ± 1.90^b^
Arginine	47.26 ± 0.68^b^	45.66 ± 0.89^b^	43.61 ± 0.97^a^	49.73 ± 0.68^b^	50.16 ± 1.03^c^	49.99 ± 0.35^c^
Non-essential amino acids (g/kg dry matter)						
Aspartic acid	86.79 ± 1.42^b^	83.10 ± 1.20^b^	73.38 ± 0.16^a^	89.45 ± 1.86^b^	89.46 ± 3.35^b^	88.77 ± 2.11^b^
Glutamic acid	127.49 ± 0.99^b^	123.11 ± 1.91^b^	110.29 ± 0.30^a^	136.95 ± 1.33^c^	139.01 ± 2.78^c^	135.84 ± 1.11^c^
Serine	35.13 ± 0.55^b^	33.58 ± 0.53^b^	31.65 ± 0.57^a^	36.64 ± 0.53^b^	36.57 ± 1.07^b^	36.50 ± 0.40^b^
Alanine	47.93 ± 0.83^b^	45.82 ± 0.74^b^	43.46 ± 0.69^a^	50.47 ± 0.68^b^	50.17 ± 1.07^b^	49.88 ± 0.66^b^
Glycine	39.78 ± 0.89^a^	38.92 ± 0.61^a^	48.91 ± 1.51^b^	43.65 ± 0.75^b^	45.56 ± 0.72^b^	44.56 ± 0.89^b^
Tyrosine	28.71 ± 0.29^b^	27.69 ± 0.64^b^	24.50 ± 0.09^a^	29.45 ± 0.59^b^	23.19 ± 1.06^a^	22.80 ± 0.61^a^
Proline	25.16 ± 0.53^b^	24.56 ± 0.52^b^	19.99 ± 0.87^a^	27.85 ± 0.10^c^	28.28 ± 0.85^c^	28.19 ± 0.61^c^
The amino acid profiles (g/kg dry matter) of the experimental diets						
Essential amino acids						
Threonine	17.62	18.54	15.33	16.42	15.11	15.81
Valine	20.14	21.86	19.34	20.19	19.04	19.25
Methionine	11.51	9.73	8.36	9.13	10.52	10.88
Isoleucine	16.85	19.16	16.36	15.88	15.47	16.43
Phenylalanine	18.16	21.94	21.18	22.26	18.02	22.48
Leucine	30.63	34.77	30.43	29.33	28.58	48.22
Histidine	13.68	13.19	11.89	13.38	11.14	10.88
Lysine	30.36	29.53	22.22	24.72	20.89	18.22
Arginine	23.23	26.97	35.4	38.12	25.92	18.18
Non-essential amino acids						
Aspartic acid	36.73	42.85	43.44	39.14	31.79	30.92
Glutamic acid	64	84.18	83.28	85.98	79.14	84.58
Serine	16.97	21.25	19.92	19.12	16.22	19.39
Alanine	25.06	22.5	21.29	21.3	21.17	29.43
Glycine	23.93	21.52	24.13	21.42	19.93	17.29
Tyrosine	13.67	17.1	16.59	14.79	14.87	18.23
Proline	25.13	26.23	21.82	21.24	20.82	30.1
ΣEAA	182.18	195.493	180.4	189.25	164.68	180.23
ΣNEAA	200.34	235.56	204.12	223.18	203.63	229.87
ΣTAA	382.58	431.29	410.98	412.32	368.39	410.12
ΣEAA/NEAA	9.13	8.23	7.82	8.42	8.12	7.85

Values are means ± SEM of 3 replications; Values within a row without a common superscript letter (a, b) are different (b indicates a higher value). EAA, NEAA, TAA, and EAA/NEAA are the abbreviation of essential amino acid, non-essential amino acid, total amino acid, and the ratio of EAA and NEAA.

### 3.4. The relative expression levels of myogenic genes in muscle

**Figure 1:**
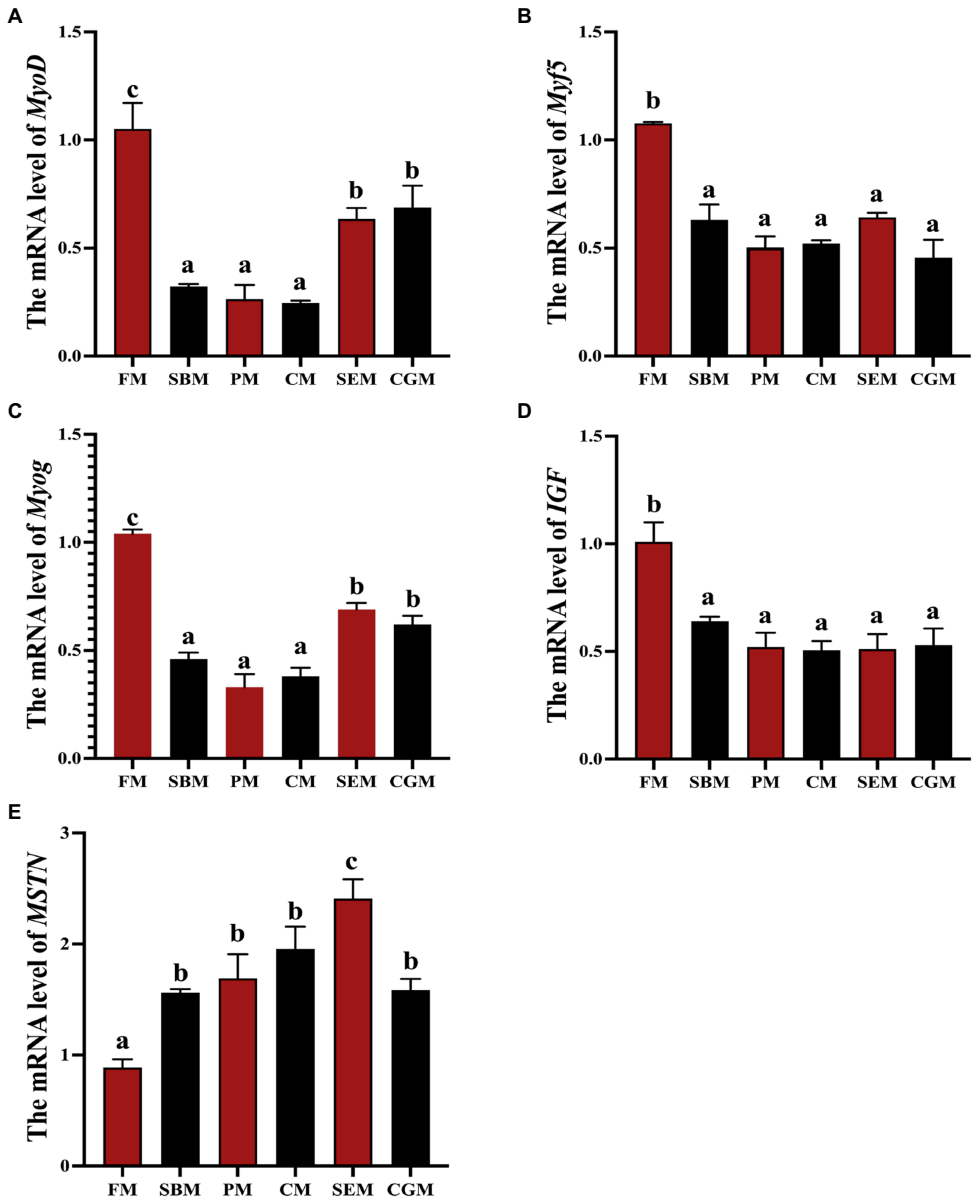
The relative expression levels of myogenic genes (*MyoD*, *Myf5*, *Myog*, *IGF*, *MSTN*) of muscle in yellow catfish **(A–E)**. Data from the FM group was set to 100% and used to normalize the remaining values. Within the figure, values without a common superscript letter (a–c) are different (c indicates a higher value). The dissimilar letters indicate significant differences (*p* < 0.05), values are means ± SEM of 3 replications.

The relative expression level of *Myod*, *Myf5, Myog, IGF-I,* and *MSTN* was shown in Figure 1. The gene expression levels of *Myod* (Figure 1A), *Myf5* (Figure 1B), *Myog* (Figure 1C), and *IGF-I* (Figure 1D) in the SBM, PM, CM, SEM, CGM were significantly lower than those in FM group (*p* < 0.05), while the gene expression level of *MSTN* (Figure 1E) was significantly increased compared with FM group (*p* < 0.05).

**Figure 2:**
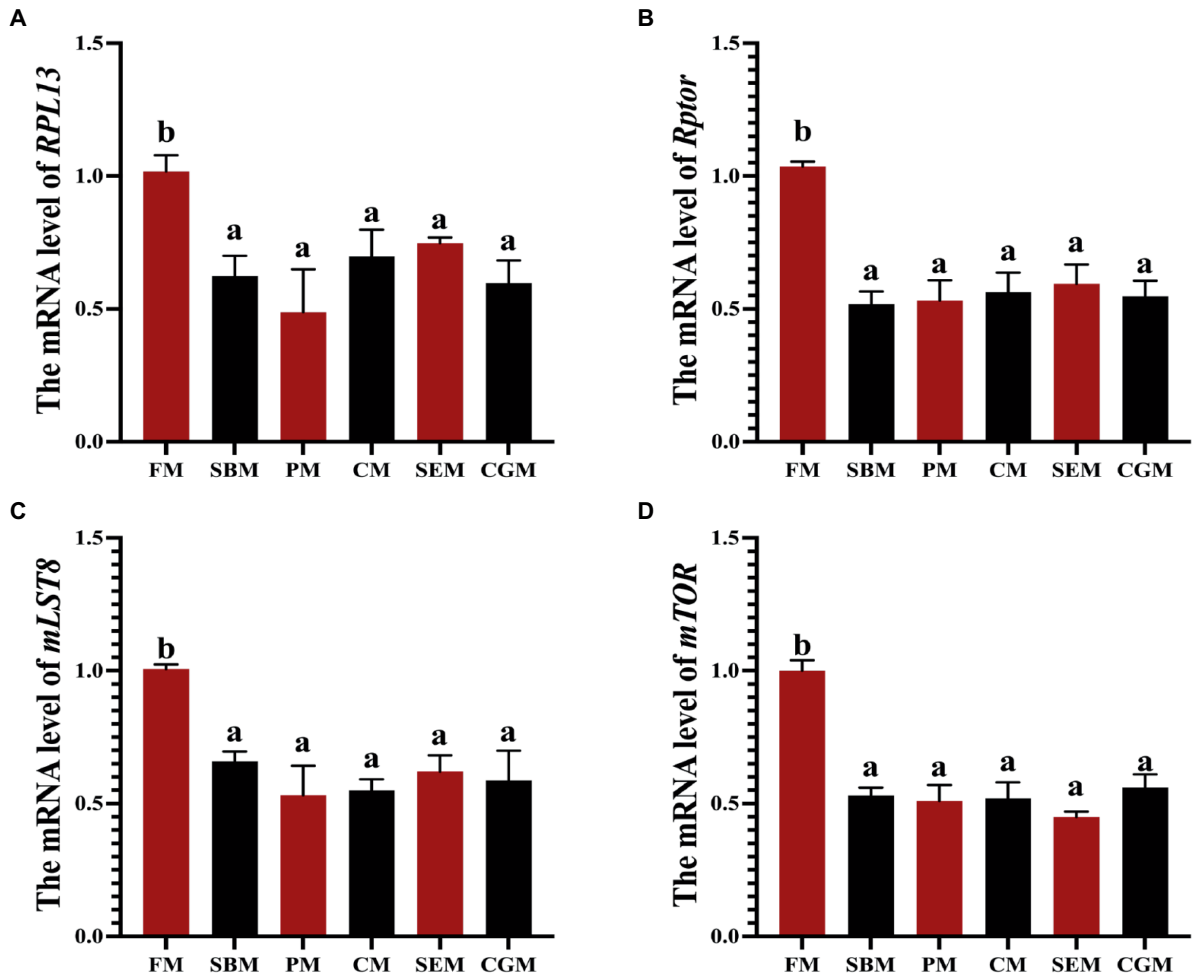
Data of mRNA levels of mTORC1 pathway genes (*RPL13, Rptor, mLST8, mTOR*) of muscle in yellow catfish **(A–D)**. The ratios of specific mRNA levels normalized to *β-actin* and *GAPDH* (used as a reference gene). Data from the FM group was set to 100% and used to normalize the remaining values. Within the figure, values without a common superscript letter (a–c) are different (c indicates a higher value). The dissimilar letters indicate significant differences (*p* < 0.05), and values are means ± SEM of 3 replications.

Figure 2 shows the relative expression levels of regulatory factors (*mTOR*, *Rptor*, *mLST8*, and *RPL13*) associated with protein synthesis. The gene expression levels of *RPL13* (Figure 2A), *Rptor* (Figure 2B), *mLST8* (Figure 2C), and *mTOR* (Figure 2D) in the SBM, PM, CM, SEM, and CGM were significantly lower than those in FM group (*p* < 0.05).

### 3.5. Muscle metabolomic analysis

**Figure 3:**
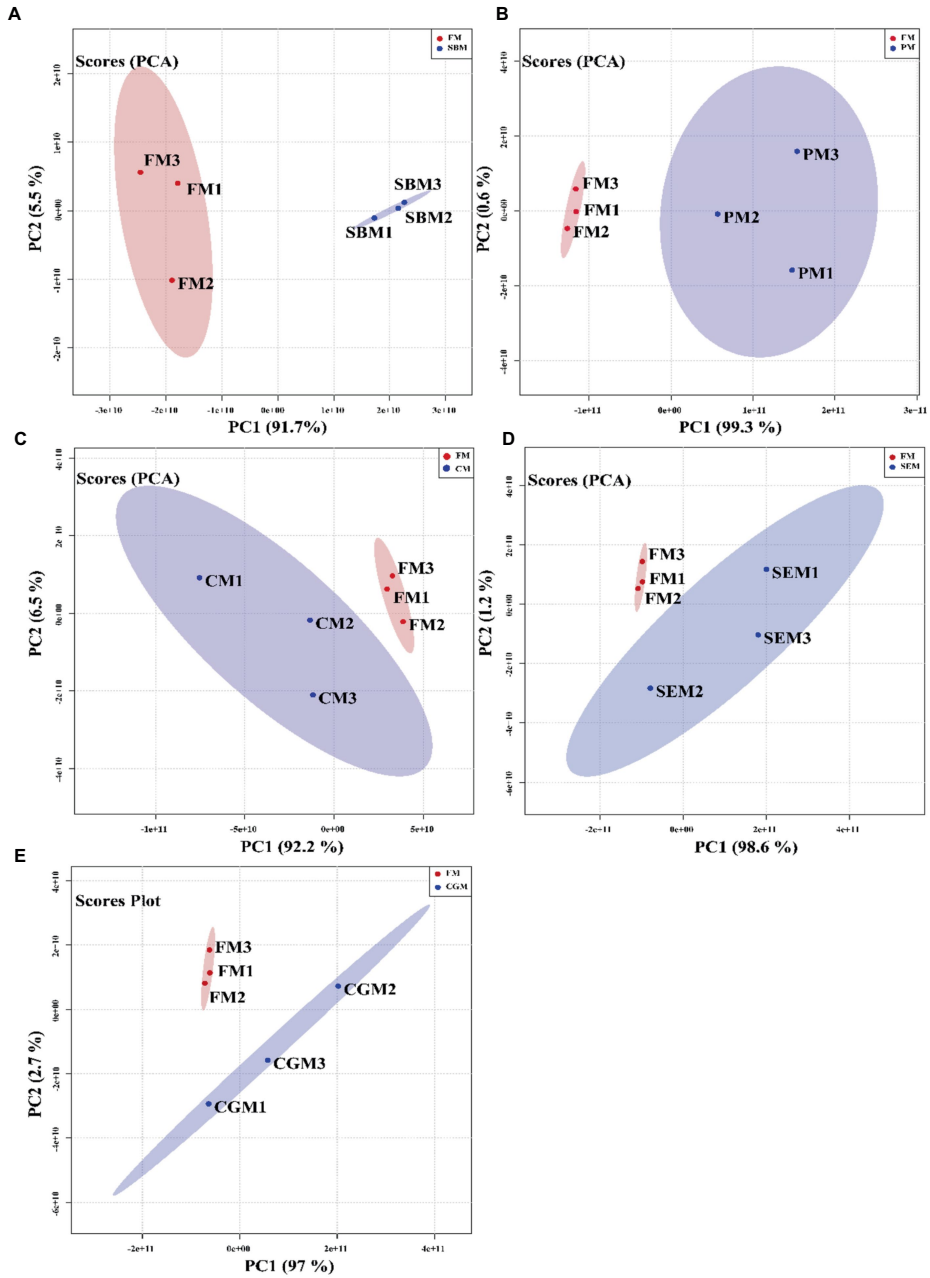
The PCA score plots for the metabolomics profiles in **(A)** FM vs. SBM; **(B)** FM vs. PM; **(C)** FM vs. CM; **(D)** FM vs. SEM; **(E)** FM vs. CGM.

The principal component analysis (PCA) score scatter plot shows that three samples in the FM group were separately clustered, and three samples in SBM, PM, CM, SEM, and CGM groups were separately clustered, and apparent segregation from the three samples in FM group (Figures 3A–E).

**Figure 4:**
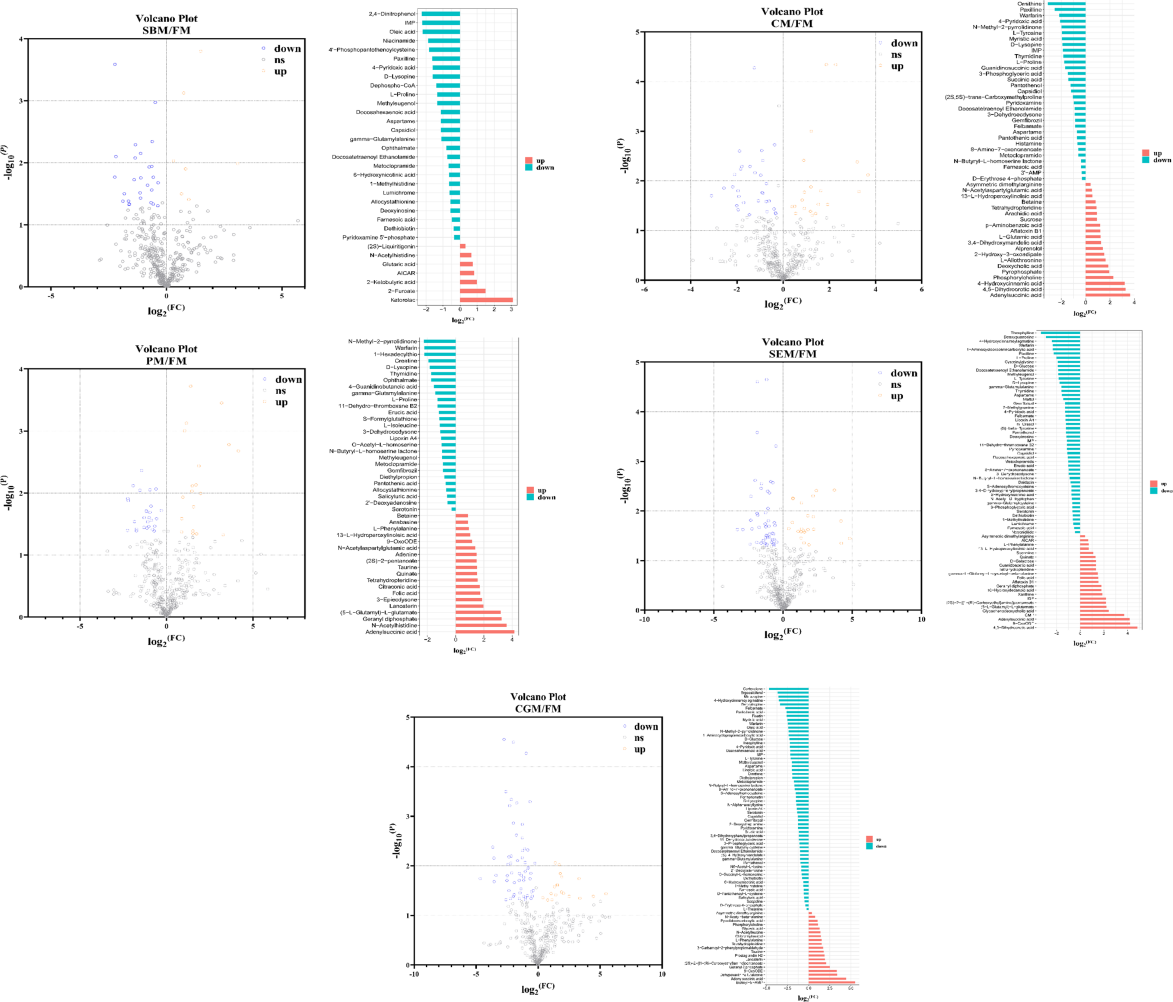
In the volcano diagram on the left, the blue circle represents the significantly down-regulated metabolites, the orange circle represents the significantly up-regulated metabolites, and the gray circle represents the metabolites that had no significant difference compared to the FM group. The bar chart on the right shows the names and fold changes of differential metabolites. The left extension represents the down-regulated metabolites, and the right extension represents the up-regulated metabolites.

As shown in the SBM/FM volcano map, a total of 33 differential metabolites (Fold change ≥ 1.2; *p* < 0.05) were detected, including 7 significantly up-regulated metabolites and 26 down-regulated metabolites (Figure 4). According to the bidirectional bar chart of SBM/FM, 2,4-Dinitrophenol, IMP and Oleic acid, and other 23 metabolites are mainly affected and significantly decreased metabolites, while 2-Ketobutyric acid, 2-Furoate, and Ketorolac and other 4 metabolites are significantly increased metabolites. In the volcanic map of PM/FM, 46 differential metabolites were detected, including 19 significantly up-regulated and 27 down-regulated. We detected 51 differential metabolites in the CM/FM group, including 31 down-regulated and 20 up-regulated differential metabolites. In the SEM/FM group, a total of 72 differential metabolites were detected, including 49 down-regulated and 23 up-regulated. In the CGM/FM group, a total of 77 differential metabolites were detected, including 58 down-regulated and 19 up-regulated.

**Figure 5:**
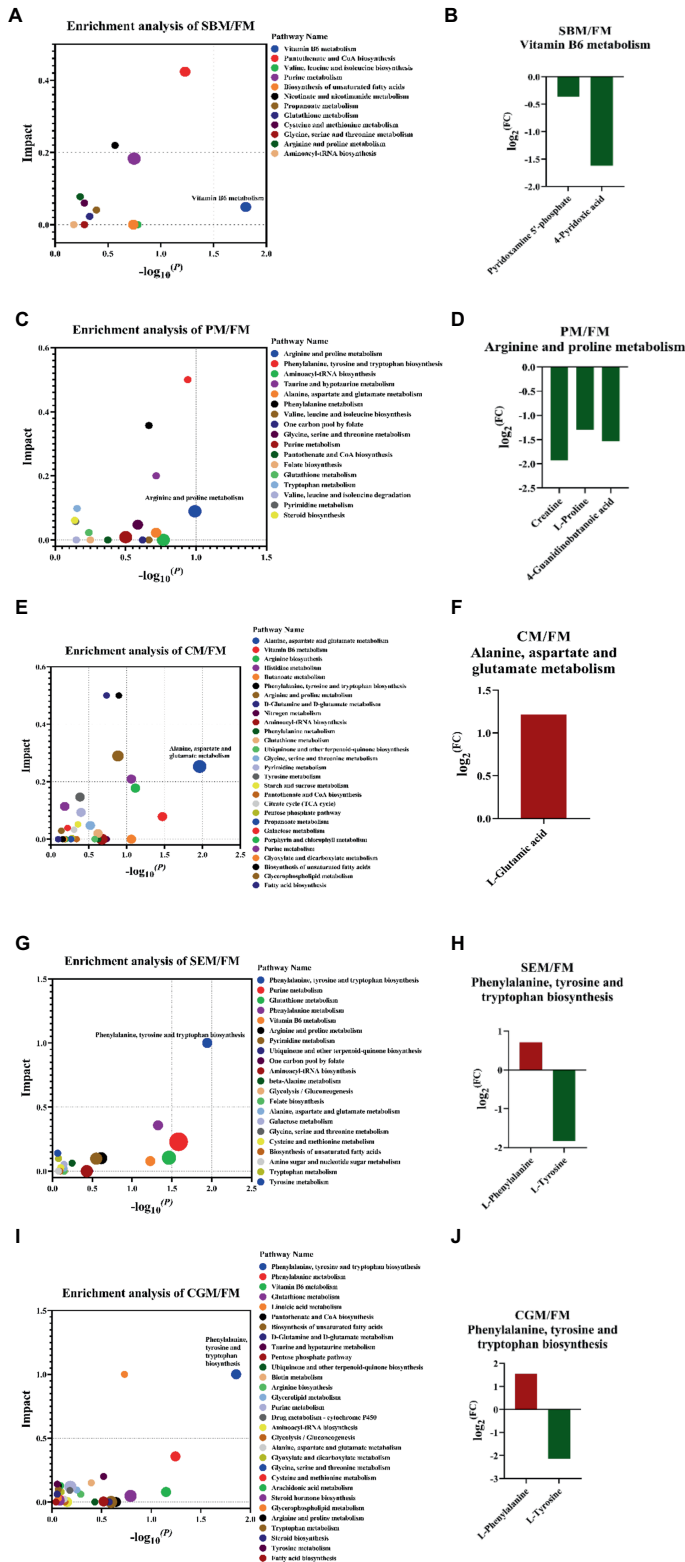
The bubble diagram exhibited the enriched KEGG pathways of differential metabolites in SBM vs. FM **(A)**, PM vs. FM **(C)**, CM vs. FM **(E)**, SEM vs. FM **(G)**, and CGM vs. FM **(I)**. Different colors represent different metabolic pathways, and the diameter of the circle represents the number of metabolites enriched in this pathway. The bidirectional bar chart exhibited the fold changes of metabolites in the most significantly affected (largest −log_10_*^P^* value) metabolic pathways **(B,D,H,F,J)**. Red columns indicate an increase in metabolites, and green columns indicate a decrease in metabolites.

In the SBM/FM group, the most affected metabolic pathway was vitamin B_6_ metabolism. In this pathway, the contents of 4-pyridoxic acid and pyridoxamine 5′-phosphate were significantly decreased in muscle (Figures 5A,B). In the PM/FM group, the most affected metabolic pathways are arginine and proline metabolism, and the contents of proline, creatine and 4-guanidinobutanoic acid in muscle are significantly decreased (Figures 5C,D). In CM/FM group, the most affected metabolic pathways are D-glutamic acid and D-glutamine metabolism as well as vitamin B_6_ metabolism. The content of glutamic acid in muscle increased significantly (Figures 5E,F). In SEM/FM (Figures 5G,H) and CGM/FM groups (Figures 5I,J), the phenylalanine, tyrosine, and tryptophan pathway were the most affected pathway. The content of phenylalanine in muscle was significantly increased while tyrosine content was significantly decreased.

### 3.6. Correlation analysis

**Figure 6:**
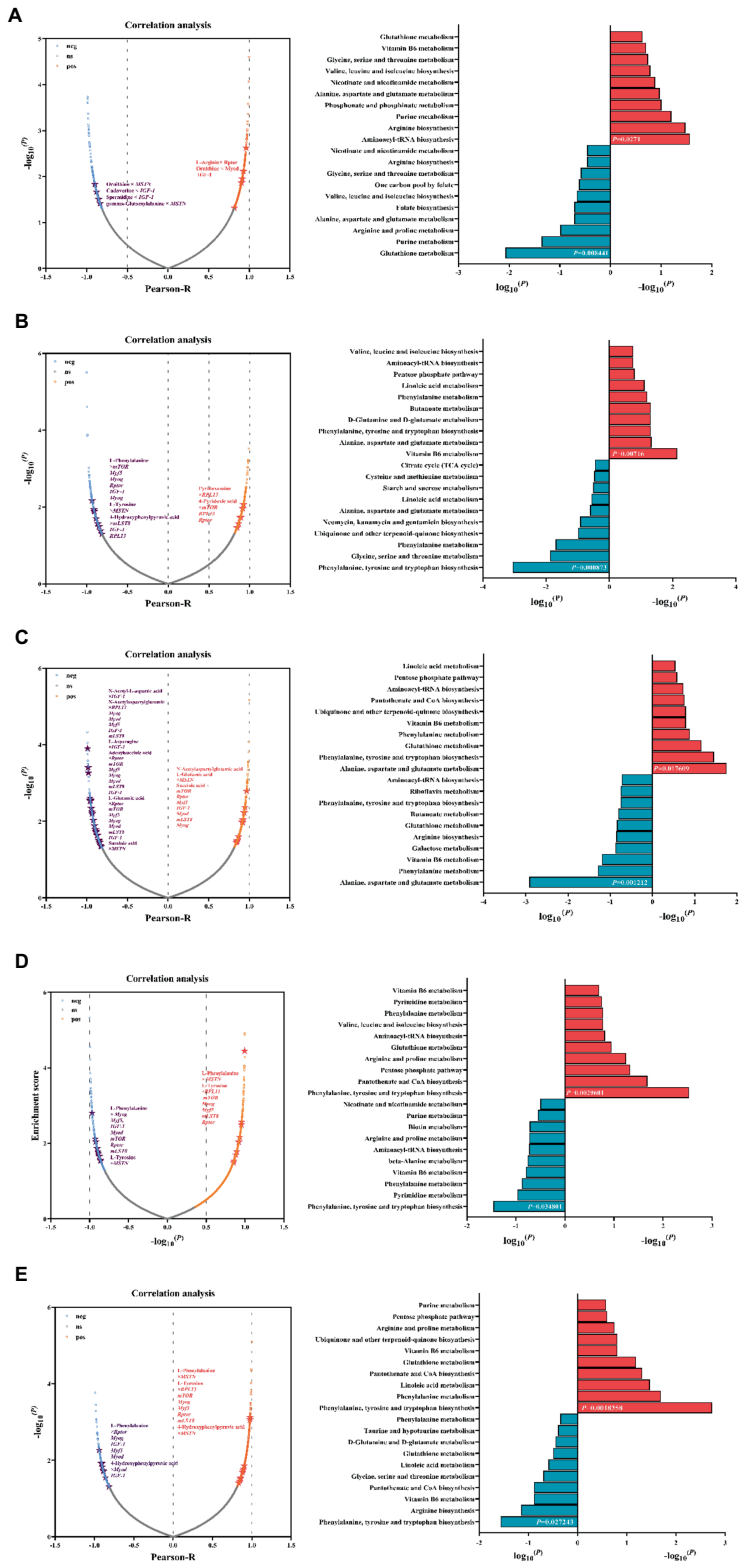
Volcano plot of the Pearson correlation between gene expression level associated with muscle proliferation and muscle metabolome of SBM vs. FM **(A)**, PM vs. FM **(B)**, CM vs. FM **(C)**, SEM vs. FM **(D)**, and CGM vs. FM **(E)**. A significant negative correlation is labeled with the blue circle (*p* < 0.05 and Pearson-R<−0.8), and a positive correlation is labeled with the orange circle (*p* < 0.05 and Pearson-R>0.8; *n* = 3 biologically independent yellow catfish). Each negatively associated metabolite and gene in the pathway (*p* < 0.05) is labeled by the purple star, and each positively associated metabolite and gene in the pathway (*p* < 0.05) is labeled by the red star. The bidirectional bar graph exhibits the top 10 enriched KEGG pathways of metabolites that are negatively and positively correlated with gene expression levels associated with muscle proliferation, respectively.

As shown in Figure 6A, each blue circle represents a pair of negative correlations between metabolites and muscle proliferation-related genes. Each orange circle represents a pair of positive correlations between metabolites and proliferation-related genes. Enrichment analysis was conducted for all metabolites negatively correlated with these genes; the results showed that the glutathione metabolism was significantly negatively correlated with muscle proliferation-related genes in the SBM/FM group (*p* < 0.05). Specifically, ornithine and gamma-glutamylalanine were negatively correlated with the mRNA level of *MSTN* (*p* < 0.05). Cadaverine and Spermidine were negatively correlated with the mRNA level of *IGF-1* (*p* < 0.05). In addition, the aminoacyl tRNA biosynthesis was positively correlated with muscle proliferation-related genes (*p* < 0.05). As shown in Figure 6B, the phenylalanine, tyrosine, and tryptophan biosynthesis were significantly negatively correlated with muscle proliferation-related genes in the PM/FM group (*p* < 0.05). Vitamin B_6_ metabolism was positively correlated with physiological indexes (*p* < 0.05). As shown in Figure 6C, in the CM/FM group, Alanine, aspartate, and glutamic acid metabolism were significantly correlated with muscle proliferation-related genes (*p* < 0.05). As shown in Figures 6D,E, in SEM/FM and CGM/FM groups, phenylalanine, tyrosine, and tryptophan biosynthesis were significantly correlated with muscle proliferation-related genes (*p* < 0.05).

## 4. Discussion

### 4.1. Growth performance and serum biochemical parameters

The data of this study showed that the growth performance and feed efficiency ratio in SBM, PM, CM, SEM, and CGM groups were significantly lower than those in the FM group. These results indicate that fish meal replacement with plant protein significantly interferes with and reduces the growth performance and feed efficiency of yellow catfish. The previous study has also shown that dietary soybean meal supplemental levels higher than 45% can induce growth retardation in groupers (*Epinephelus coioides*) (31). A high substitution level of peanut meal can significantly decrease the expression of antimicrobial peptide genes, significantly increase the abundance of intestinal pathogenic bacteria, and decrease the abundance of beneficial bacteria in juvenile hybrid grouper (*Epinephelus fuscoguttatus ♀ × Epinephelus lanceolatus ♂*) (32). Substitution of 45% or more cottonseed meal for soybean meal had adverse effects on the intestinal health and growth performance of Nile tilapia (*Oreochromis niloticus*) (33). In addition, the substitution of 45% sesame meal and 50% corn gluten meal can decrease the growth performance and feed efficiency ratio of European sea bass (10). In conclusion, dietary supplementation of 50% SBM, PM, CM, SEM and CGM can adversely affect the growth performance of yellow catfish. Previous studies reported that the HDL transferred lipids from peripheral tissues to the liver for catabolism, which helps decrease lipid accumulation in fish (34) and the LDL transferred cholesterol from the inside of the liver to peripheral tissues (35). In this study, the HDL content in the PM, CM, and SEM groups was significantly higher than that in the FM group, while the LDL content in SEM and CGM groups was significantly lower in the FM group. The occurrence of this condition indicates an increase occurred in cholesterol flow into the liver from the peripheral environment and a decrease occurred in cholesterol outflow from the liver (36, 37). Correspondingly, the data of this study also evidenced that the serum cholesterol level of the PM, CM, SEM, and CGM groups was significantly higher than that of the FM group. This suggests that plant protein components may induce a type of liver impairment that inhibits the ability of liver lipid decomposition.

### 4.2. Whole-body proximate composition, and muscle amino acid

In general, the protein content is one of the crucial indexes to evaluate the nutritional value and flesh quality of muscle (18). It was found that the crude protein content in CM, SEM, and CGM group was significantly lower than that in the FM group, indicating that the nutritional value of CM, SEM, and CGM was inferior to FM. Aspartate, glutamic acid, alanine, proline, threonine, glycine, and serine as flavor compounds also play an important role in the taste and flavor of fish (38). Partial substitution of fish meal with PM, SEM, CM, and CGM can significantly increase the contents of these flavor amino acids (glutamic acid, glycine, and proline) in fish muscle, which has a positive effect on the improvement of the flavor of yellow catfish. A previous study has shown that arginine (Arg) can improve shear force and firmness of muscle, thus improving the flesh quality of grass carp (*Ctenopharyngodon idella*) (12, 38, 39). Similarly, in this study, partial substitution of fish meal with sesame meal and corn gluten meal increased Arg content in muscle, and the hardness, springiness, chewiness, and gumminess were significantly increased in SEM, CM, and CGM groups compared with the FM group. These results showed that arginine had a positive effect on the flesh quality of yellow catfish. It is noteworthy that only the compositions of glycine in PM were significantly increased, while the compositions of other amino acids were significantly lower than in the FM group, suggesting that the nutritional value of peanut meal was significantly inferior to fish meal.

### 4.3. Determination of myogenic regulatory factors expression

The proliferation and differentiation of myogenic progenitor cells (MPCs) that were responsible for muscle development and regeneration were highly correlated with the increase in the number of small-diameter muscle fibers (18). Meanwhile, the expression of myogenic regulatory factors (MRFs) such as *Myf5*, *Myod*, *Myog*, and *Mrf5* was a significant feature of the proliferation and differentiation of muscle cells. The up regulation of *Myog*, *MyoD*, and *Mrf5* can promote hyperplasia, inflation, and growth of muscle (18). Therefore, the relative gene expression levels of the MRFs gene and its crucial positive and negative regulatory signals (*IGF-I* and *MSTN*) were analyzed by q-PCR in this study. Compared with FM, transcription of *Myod*, *Mrf5*, *Myog*, and *IGF-I* was significantly down-regulated and *MSTN* was significantly up-regulated in SBM, PM, CM, SEM, and CGM groups. These data suggest that plant protein components may inhibit muscle growth by inhibiting the proliferation and differentiation of MPCs by influencing myogenic-related signaling pathways. The evolutionarily conserved target of rapamycin complex 1 (*mTORC1*) is a central regulator of cell growth and metabolism that was highly sensitive to protein synthesis regulators. A recent study has shown that the expression of *mTOR* mRNA levels in *Megalobrama amblycephala* is affected by nutritional factors (40). The *mTORC1* complexes are composed of *mTOR*, *Raptor* (regulatory-associated protein of mTOR), The gene composition of *RPL13* (ribosomal protein L13), and m*LST8* (mammalian lethal with SEC13 protein 8), *mTORC1* play a crucial role in the regulation of muscle growth which is also in charge of the regulation of muscle proliferation (41). In this study, the mRNA levels of *mTOR*, *mLST8*, and *Raptor* genes in SBM, PM, CM, SEM, and CGM groups were significantly decreased, suggesting that the plant protein ingredients inhibited the molecular signals (*mTORC1*) of muscle growth and development. This may be induced by the unbalanced amino acid composition of plant proteins, as a recent study has shown that crystal amino acid supplementation can promote protein synthesis in the muscle of Nile Tilapia (*Oreochromis niloticus*) by regulating the expression of genes related to the *mTORC1* signaling pathway (42).

### 4.4. Metabolomics analysis

Metabolomics can be utilized successfully to detect variations in metabolites and to ascertain the effects of dietary interventions on metabolic pathways through enrichment analysis of differential metabolites (18). KEGG enrichment analysis showed that soybean meal primarily affected vitamin B_6_ (VB_6_) metabolism. The precursors (pyridoxamine 5′-phosphate) and downstream metabolites (4-pyridoxic acid) of VB_6_ in the SBM group were significantly lower than in the FM group. This suggests that the utilization efficiency of VB_6_ may be improved. VB_6_ acts as a prosthetic group for enzymes in a wide range of metabolic reactions, particularly those related to the anabolism of amino acids (43). In the present study, free proline and aspartic acid contents were reduced in muscle, suggesting that VB_6_ metabolism participates in protein biosynthesis in muscle by recruiting free amino acids in muscle. Therefore, soybean meal and fish meal groups had similar muscle amino acid composition and crude protein content. PM mainly affects proline metabolism. Proline is a functional amino acid involved in collagen production which is also involved in the synthesis of protein (44). The metabolic data showed that the content of proline in muscle was decreased. Meanwhile, the proportion of proline and the crude protein content in muscle was significantly lower than in the FM group. The reasonable explanation is that the proline content in peanut meal could not meet the nutritional requirements of yellow catfish. Moreover, the congenital deficiency of endogenous synthesis of proline is ubiquitous in fish (45). Therefore, peanut meal intake can aggravate the proline deficiency in muscle and further reduce the substrate for muscle protein synthesis, which leads to a decrease in both crude protein content and proline ratio in muscle. The previous study has demonstrated that proline deficiency in feed impairs growth performance and crude protein content of juvenile Spotted drum (*Nibea Diacanthus*) (45). CM mainly affects glutamic acid metabolism. Glutamic acid occupies a central position in the biosynthesis of amino acids. Glutamic acid can be converted to α -ketoglutaric acid and other amino acids with the mediation of different transaminases and glutamic acid dehydrogenases (46). Metabolomics data showed that glutamic acid content in muscle was significantly higher than in the FM group. This was consistent with the amino acid composition analysis of muscle in the present study. The present study concluded that this is probably due to the abnormal accumulation of glutamic acid caused by cottonseed meal and resulting in the disturbance of protein biosynthesis in muscle, which resulted in lower crude protein content in the CM group than in the FM group. In contrast, optimal glutamic acid intake and higher glutamic acid utilization significantly increased protein retention in the muscle of juvenile gilt-head bream (*Sparus Aurata*) (47). SEM and CGM mainly affect phenylalanine and tyrosine metabolism. Phenylalanine is a precursor of tyrosine biosynthesis (48). Metabolome data showed that the phenylalanine content in muscle was significantly higher than the FM group while the tyrosine content was significantly lower than the FM group which was consistent with amino acid composition analysis. We hypothesize that the conversion of phenylalanine to tyrosine is inhibited, resulting in the accumulation of phenylalanine and the deficiency of tyrosine. Tyrosine is indispensable for the growth and development of fish and the decrease in tyrosine content may be one of the reasons for the decrease in crude protein content. In this study, the reduction of crude protein content in SEM and CGM groups indicates that protein synthesis is impeded due to tyrosine deficiency in muscles. Previous studies have also shown that adequate supplementation of tyrosine can optimize growth performance, body protein synthesis, and other physiological functions of Catla Catla (*Hamilton*) (49).

### 4.5. Correlation analysis between metabolome and MRFs

In the CM/FM group, the glutamic acid was negatively correlated with gene expression levels involved in muscle proliferation (*Rptor*, *mTOR*, *Myf5*, *Myog*, *Myod*, *mLST-8*, and *IGF-1*). This suggests that the inhibition of myocyte proliferation genes may be related to the accumulation of glutamic acid in muscle. Glutamic acid can be converted to glutamine, and supplementation of dietary glutamine in Atlantic salmon can promote the proliferation of muscle cells (50). We hypothesize that the interruption of the glutamic acid-to-glutamine pathway results in the loss of stimulatory signals for genes involved in muscle proliferation, and thus gene expression level is repressed. In SEM/FM, and CGM/FM groups, tyrosine was positively correlated with *Myog*, *Myf5*, and *mTOR* expression, suggesting that the expression of muscle proliferation-related genes may be regulated by tyrosine. Tyrosine is a precursor of thyroid hormone, which significantly promotes muscle development and regeneration (51). We hypothesized that the reduction of tyrosine in muscle leads to the inhibition of biosynthesis of thyroid hormone that promotes myocyte proliferation, and the gene expression involved in muscle proliferation was also depressed. The results of correlation analysis showed that the inhibition of different plant proteins on muscle proliferation and differentiation was mainly achieved by disrupting different amino acid metabolism to interfere with the expression of muscle growth-related genes, which may be caused by the imbalance of amino acid composition and the presence of antinutritional factors in plant proteins.

## 5. Conclusion

Utilizing CM, SEM, and CGM as part of the protein source can improve the fillet texture parameters of fish, especially the fillet hardness and chewiness. However, due to the imbalance of amino acid composition and the presence of anti-nutritional factors, the advantages of CM, SEM, and CGM in flesh quality must be discreetly evaluated. In the actual production of aquaculture, cultured yellow catfish fed with CM, SEM, and CGM can be regarded as having the positive potential to improve flesh quality, flavor, and taste. Nevertheless, CM, SEM, and CGM had significant negative effects on growth performance and physiological state. SBM, PM, CM, and SEM (CGM) regulated the biosynthesis and degradation of muscle protein by affecting the content of vitamin B6, proline, glutamic acid, and phenylalanine (tyrosine) in muscle, thus affecting the flesh quality. Meanwhile, the inhibition of myocyte proliferation-related genes in CM, SEM, and CGM groups may also be regulated by the increase of glutamic acid content and the decrease of tyrosine content.

## Data availability statement

The original contributions presented in the study are included in the article/supplementary material, further inquiries can be directed to the corresponding author.

## Ethics statement

All management conditions and experimental protocols were approved by the Animal Care Advisory Committee of Ningbo University and conducted in accordance with the Guidelines for the Care and Use of Laboratory Animals of Ningbo University under permit SYXK (ZHE 2012-011012). All efforts were made to minimize the suffering of yellow catfish.

## Author contributions

XL, SW, and MZ carried out the experimental work. XL wrote the manuscript under the direction of RW and YQ. HJ helped to revise the manuscript. ML conceived of the study. All authors contributed to the article and approved the submitted version.

## Funding

This work was supported by the National Natural Science Foundation of China (32072948, 32202908, and 32260912); the Fundamental Research Funds for the Provincial Universities of Zhejiang (SJLY2020009); the Natural Science Foundation of Ningbo City (202003N412); the Natural Science Foundation of Guizhou Province of China (ZK2022-145); the Natural Science Research Fund (Special-post) of Guizhou University (2021-26).

## Conflict of interest

The authors declare that the research was conducted in the absence of any commercial or financial relationships that could be construed as a potential conflict of interest.

## Publisher’s note

All claims expressed in this article are solely those of the authors and do not necessarily represent those of their affiliated organizations, or those of the publisher, the editors and the reviewers. Any product that may be evaluated in this article, or claim that may be made by its manufacturer, is not guaranteed or endorsed by the publisher.

## Footnotes

^1^https://www.metaboanalyst.ca/
